# Sensitivity of PM_10_ oxidative potential to aerosol chemical composition at a Mediterranean urban site: ascorbic acid versus dithiothreitol measurements

**DOI:** 10.1007/s11869-023-01332-1

**Published:** 2023-03-13

**Authors:** Á. Clemente, J. Gil-Moltó, E. Yubero, N. Juárez, J. F. Nicolás, J. Crespo, N. Galindo

**Affiliations:** Atmospheric Pollution Laboratory (LCA), Department of Applied Physics, Miguel Hernández University, Avenida de La Universidad S/N, 03202 Elche, Spain

**Keywords:** PM_10_, Oxidative potential, Chemical components, Seasonal variations, Correlation analysis

## Abstract

The oxidative potential (OP) of PM_10_ daily samples collected at a traffic site in southeastern Spain during summer and winter was assessed by two acellular assays: the ascorbic acid (AA) and dithiothreitol (DTT) methods. Although PM_10_ levels were similar during both periods, OP values (expressed in nmol min^−1^ m^−3^) showed a defined seasonal trend. The AA activity was higher in summer than in winter, whereas the DTT reactivity exhibited an opposite seasonal pattern. Both assays were sensitive to different PM_10_ components, as shown by the results of the linear correlation analysis. Moreover, the relationship between OP values and PM_10_ chemical species was not the same during summer and winter, indicating that particle toxicity is associated with different sources during the warm and cold seasons. When OP values were expressed on a mass basis (nmol min^−1^ µg^−1^), lower correlation coefficients with PM_10_ chemical species were generally obtained compared to volume-normalized activities. These outcomes suggest that only some specific components have a significant intrinsic oxidative potential.

**Supplementary Information:**

The online version contains supplementary material available at 10.1007/s11869-023-01332-1.

## Introduction 

Exposure to airborne particulate matter (PM) has been unequivocally linked to various adverse effects on human health, including cardiopulmonary and cerebrovascular diseases, lung cancer, aggravated allergies, and birth defects (Bodor et al. 2022; Contini et al. 2021; Zhang et al. 2022). The severity of the health outcomes depend upon particle size and chemical composition (Kim et al. 2015). Most of the particle mass comprises low toxicity components such as ammonium sulfate and nitrate, sodium chloride, and mineral dust (Ayres et al. 2008). In contrast, low levels of components such as transition metals and water-soluble organic compounds can induce severe health effects since they are potent oxidants, i.e., are able to generate excessive reactive oxygen species (ROS) (Borm et al. 2007; Saffari et al. 2014). Oxidative stress results when the generation of ROS exceeds the available antioxidant defenses and can cause different biological processes such as inflammation and cell damage (Andrés Juan et al. 2021; Lodovici and Bigagli 2011). The oxidative potential (OP) is a measure of the capacity of PM to oxidize target molecules and is considered a better metric of PM toxicity than PM mass alone (Calas et al. 2018; Molina et al. 2020). Recent works have shown that cytotoxic and ecotoxic effects measured using in vitro tests depend on PM chemical composition rather than on mass concentrations, and that cytotoxicity is correlated with acellular OP measurements, although the strength of the correlation may display seasonal and site-to-site variations (Guascito et al. 2023; Lionetto et al. 2019).

Among the methods developed to measure the OP in PM samples, the dithiothreitol (DTT) and ascorbic acid (AA) acellular assays are widely used. These assays have the advantages of being fast and using low-cost spectrophotometric measurements (Bates et al. 2019; Pietrogrande et al. 2019). OP is assessed from the consumption rate of AA (OP^AA^), used as proxy of endogenous antioxidant species, or the depletion of DTT (OP^DTT^), a reductant surrogate, in PM extracts (Gao et al. 2020). Different OP methods are sensitive to different PM chemical components. In general, OP^AA^ is especially sensitive to metals such as copper and iron (Bates et al. 2019; Janssen et al. 2014; Massimi et al. 2020), while OP^DTT^ is more responsive to copper, manganese, and organic species (Bates et al. 2019; Fang et al. 2016; Visentin et al. 2016). In spite of this, responses of OP assays to the chemical composition of PM vary significantly depending on the sampling site and season of the year (Fang et al. 2016; Gao et al. 2020).

This study is aimed at assessing the seasonal variability of OP^AA^ and OP^DTT^ in PM_10_ samples collected at a typical Mediterranean urban site, as well as examining the sensitivity of both assays to different PM chemical species in order to provide insights into the influence of chemical composition on PM toxicity.

## Materials and methods

### Sampling site and PM_10 _measurements

PM_10_ samples were collected for 24 h, three times a week, in the city center of Elche (southeastern Spain, less than 15 km from the Mediterranean coast). The sampling site was located on the first floor of a building on one side of a 7-m-wide street. A Derenda 3.1 low volume sampler (2.3 m^3^ h^−1^) with a PM_10_ inlet was used for sample collection onto quartz fiber filters. The sampling period was from December 2020 to February 2021 (winter) and from June to August 2021 (summer). A total of 30 PM_10_ samples per season were analyzed to determine the chemical composition and oxidative potential.

Gravimetric mass was measured using an Ohaus AP250D analytical balance. Filters were weighed after a 24-h conditioning at 20 ± 1ºC and 50 ± 5% relative humidity. More details on the sampling site and the measurement procedure can be found in Galindo et al. (2018a) and Clemente et al. (2022).

### Chemical composition analysis

Energy dispersive X-ray fluorescence (ED-XRF) was used to determine the elemental composition of PM_10_ samples by means of an ARL Quant’x Spectrometer (Thermo Fisher Scientific, UK) with a Si(Li) detector. A detailed description of the analytical technique can be found elsewhere (Chiari et al. 2017).

After the elemental analysis, 1.5 cm^2^ of each filter was used to determine elemental carbon (EC) and organic carbon (OC) concentrations by means of a thermal-optical transmission (TOT) analyzer (Sunset Laboratory, Inc.) using the EUSAAR-2 protocol.

Separate portions of each filter were extracted ultrasonically with ultrapure water and analyzed by ion chromatography (1/2 of each filter—1.5 cm^2^) and high-performance anion-exchange chromatography coupled with pulsed amperometric detection (1/4 of each filter) to determine major ions (Cl^−^, NO_3_^−^, SO_4_^2−^, C_2_O_4_^2−^, Na^+^, NH_4_^+^, Mg^2+^, K^+^, and Ca^2+^) and levoglucosan, respectively. The analytical methods used for the determination of anions and cations are described in Galindo et al. (2018b).

The analysis of levoglucosan was performed by means of a Thermo Scientific Dionex Integrion system equipped with an electrochemical detector and a Dionex Carbopac PA10 analytical column (250 × 4 mm). NaOH was used as a carrier solvent at a flow rate of 0.5 ml min^−1^. The gradient was: 18 mM (0–2 min), 200 mM (2–9 min; column cleaning), 18 mM (9–29 min; equilibration). For the amperometric detection, a gold working electrode was used.

### Oxidative potential assays

A quarter of each filter was extracted with 7 mL of ultrapure water for 30 min in an ultrasonic bath. Extracts were then filtered using nylon syringe filters (0.45 µm) to remove insoluble material and analyzed by both the DTT and AA acellular methods.

In the AA assay, aliquots of 1.5 mL of the sample extracts were incubated with 1.35 mL of 0.1 M potassium phosphate buffer (pH 7.4) and 150 µL of 2 mM AA at 37ºC. After the addition of ascorbic acid, the absorption at 265 nm was measured at known time intervals to determine the rate of AA depletion.

For the DTT assay, a similar procedure to that described in Massimi et al. (2020) was applied. Three aliquots of 0.45 mL of the extracts were incubated at 37ºC with 90 µL of 0.1 M potassium phosphate buffer (pH 7.4) and 60 µL of 1 mM DTT. After an incubation period of 15, 25, and 35 min, respectively, 0.5 mL of trichloroacetic acid (10% w/v) were added to each one of the aliquots to stop the reaction. Then, 2 mL of Tris–EDTA (0.4 M Tris with 20 mM EDTA) and 50 µL of 10 mM 5,5′-dithiobis-2-nitrobenzoic acid (DTNB) were added to the mixture and the absorbance of the solution was recorded at 412 nm.

In both methods, the initial concentration of the antioxidant was 100 nmol mL^−1^. Blank filters were analyzed following the same procedures as those of PM_10_ samples. All assays were run in duplicate. OP^AA^ and OP^DTT^ values normalized per cubic meter (nmol min^−1^ m^−3^), representative of human exposure, were calculated from AA and DTT depletion rates, respectively. Mass normalized OP activities (nmol min^−1^ µg^−1^) were also determined.

## Results and discussion

### Seasonal variability of the oxidative potential

**Fig. 1:**
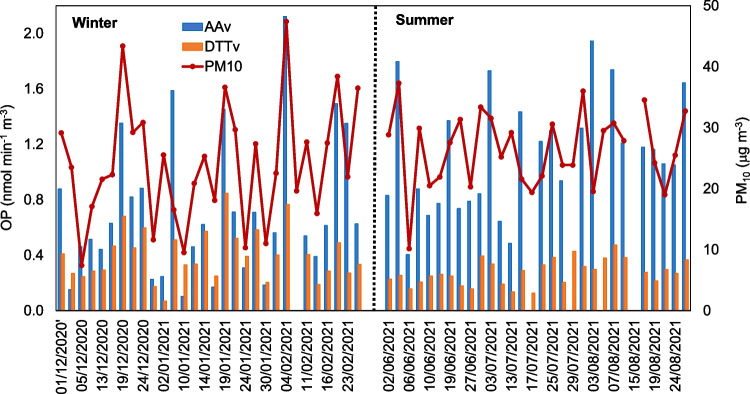
Daily variability of PM_10_, OP^AA^, and OP^DTT^ during winter and summer

Seasonal and average OP and PM_10_ values measured in Elche during the study period are presented in Table 1. Figure 1 displays time series of PM_10_ levels and OP activities during the summer and winter campaigns. Mean concentrations of PM_10_ chemical components during the cold and warm seasons are reported in Table S1 of the Supplementary Material.

**Table 1:** PM_10_ concentrations (µg m^−3^) and OP values (nmol min^−1^ m^−3^) averaged for the whole period, summer and winter (± standard deviation). Mean values for some meteorological parameters are also included

	Mean	Winter	Summer	% Difference^a^
PM_10_	25.3 ± 8.3	24.2 ± 9.9	26.5 ± 6.2	9.5
*OP^AA^	0.91 ± 0.51	0.71 ± 0.50	1.12 ± 0.42	57.7
*OP^DTT^	0.34 ± 0.16	0.40 ± 0.18	0.28 ± 0.09	− 30.0
*T (ºC)	19.9 ± 6.7	13.9 ± 3.4	26.0 ± 2.1	46.5
*Solar rad. (W m^–2^)	178 ± 96	104 ± 35	253 ± 79	59.9
*RH (%)	71 ± 14	67 ± 16	75 ± 11	10.7

*Differences between summer and winter averages were statistically significant (Student’s *t*-test, *p* < 0.05).

^a^Percentage difference between summer and winter values were calculated as follows: Difference (%) = [(summer average – winter average)/winter average] × 100.

PM_10_ concentrations were similar during winter and summer, as previously reported for the same sampling site (Galindo et al. 2020). The temporal variability of PM levels mainly depends on emissions from sources and meteorological conditions. During summer, the stronger solar radiation results in a higher formation of secondary aerosols by photochemical reactions. Besides, the lower rainfall favors local resuspension of road dust particles. In contrast, the poor dispersion conditions and lower temperatures during the cold season promote the accumulation of pollutants emitted by local sources and the condensation of semi-volatile species to the aerosol phase, respectively. The above factors, which are common in the Mediterranean region, may explain the absence of a clear seasonal trend during the measurement period. On the other hand, the influence of sources such as biomass burning, which contribute to higher winter PM_10_ concentrations at other locations (e.g., Borlaza et al. 2021), is low in the study area (Galindo et al. 2021).

PM_10_ average levels found in the present study were slightly lower than those previously measured at the same site (~ 28 µg m^−3^ during both summer and winter, Galindo et al. 2020). A possible reason for this outcome is that during the sampling period some restrictive measures due to the COVID-19 pandemic were still in place. Therefore, the results of the OP assays could have been affected by a certain reduction in anthropogenic emissions during the measurement period.

OP average values, especially for the AA assay, were higher than those registered at a suburban site in the central Mediterranean (OP^DTT^ = 0.24 nmol min^−1^ m^−3^, OP^AA^ = 0.29 nmol min^−1^ m^−3^; Pietrogrande et al. 2018), despite the average PM_10_ concentration at this location being greater (33 µg m^−3^) than that measured in Elche. In contrast, the OP values obtained in the present work were significantly lower than those reported for the city of Chamonix (French Alps; Calas et al. 2018), where average PM_10_ levels in winter were similar to the values found in this study (29 µg m^−3^) while the average concentration during the warm season (10 µg m^−3^) was less than half that observed in Elche. These results suggest, as indicated in previous works (Lionetto et al. 2019), that PM mass alone cannot adequately assess aerosol toxicity. In fact, at the sampling site in Elche moderate correlations of PM_10_ concentrations with OP^AA^ and OP^DTT^ were found (Table 2), contrary to the results of other studies (Calas et al. 2018, *r* > 0.8 for both assays; Janssen et al. 2014, *r* = 0.75 for OP^DTT^). Correlations were stronger in winter (*r* ≈ 0.68) than in summer (*r* ≈ 0.38) for both assays, in line with previous research (Calas et al. 2018; Campbell et al. 2021; Perrone et al. 2019). This might be explained by higher collinearity between concentrations of PM_10_ components and overall PM_10_ mass concentrations during winter (data not shown) likely due to meteorological conditions.

**Table 2:** Pearson correlation coefficients among OP values and PM_10_ concentrations

	PM_10_		
	Whole period	Winter	Summer
OP^AA^	0.59	0.69	0.37
OP^DTT^	0.49	0.68	0.38

The DTT reactivity was found to be higher during the cold season, while AA activity showed an opposite seasonal trend, with lower levels in winter than in summer. An equivalent seasonal variability has been observed in previous studies (Fang et al. 2016) and has been attributed to differences in the sources for OP^AA^ and OP^DTT^. However, these results contrast with those obtained in other works. For instance, Calas et al. (2018), Campbell et al. (2021), and Borlaza et al. (2021) reported higher OP values during winter for both the AA and DTT assays due to the larger contribution of PM components from biomass burning emissions. Giannossa et al. (2022) also obtained a higher OP^DTT^ in PM_2.5_ during wintertime, which they attributed to the association of the DTT activity with local combustion sources (biomass burning and traffic) that likely have a greater influence on the DTT than the AA response. On the other hand, Pietrogrande et al. (2018) found similar values during the cold and warm periods for both assays. The differences between the seasonal behavior of OP values among different locations could be attributed to the inter site variability in the concentrations of PM redox-active species (Pietrogrande et al. 2018). However, it should also be considered that synergistic and antagonistic interactions between PM components may affect OP values (Borlaza et al. 2021; Pietrogrande et al. 2022) and that these interactions depend on the aerosol chemical composition, which is characteristic of the monitoring site. It is interesting to point out that both the OP^AA^ and OP^DTT^ were more variable during winter than summer, similarly to previous studies (Calas et al. 2018).

**Fig. 2:**
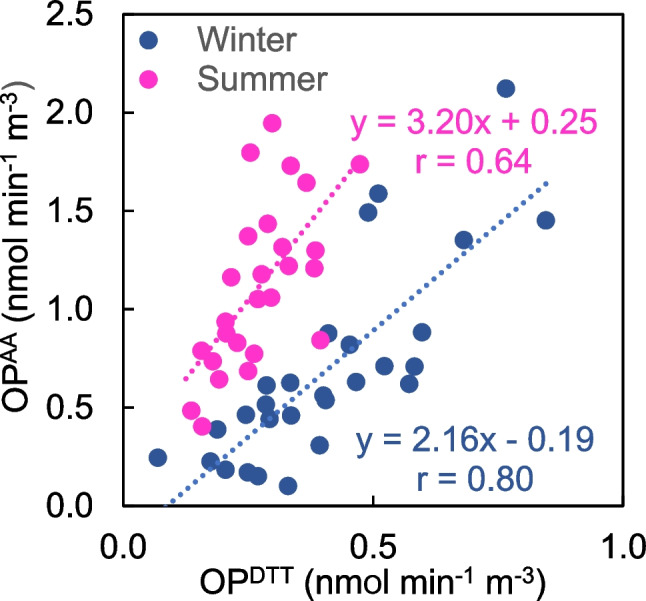
Relationship between OP^AA^ and OP^DTT^ during winter and summer

As expected considering their opposite seasonal cycle, OP^AA^ and OP^DTT^ were poorly correlated (*r* = 0.4). However, when correlations were analyzed separately for the cold and warm periods, better correlation coefficients were found (Fig. 2). This may be related to differences in the PM_10_ chemical composition during summer and winter.

### Correlation of the oxidative potential with PM_10_ chemical components

In order to gain insights into the possible sources for OP^AA^ and OP^DTT^, a correlation analysis between both assays and the chemical species analyzed in PM_10_ was performed. The relationship between OP values and chemical components was assessed on the basis of linear regressions (Pearson’s *r*). The results for the winter and summer seasons are presented in Table 3. Correlations were considered high for *r* ≥ 0.7 and moderate for *r* between 0.45 and 0.70, according to the criteria of previous works (Calas et al. 2018; Farahani et al. 2022; Janssen et al. 2014).

**Table 3:** Pearson correlation coefficients among OP measurements and PM_10_ chemical components

	OP^AA^			OP^DTT^	
	Winter	Summer		Winter	Summer
K	0.51	0.18		**0.80**	0.39
Ti	0.52	− 0.18		0.55	0.31
Mn	0.20	0.30		0.45	0.07
Fe	0.35	0.27		**0.71**	0.41
Cu	0.35	**0.78**		0.69	0.47
Zn	0.37	− 0.19		**0.72**	− 0.42
Cl^−^	0.03	− 0.09		− 0.06	− 0.25
NO_3_^–^	**0.83**	0.35		0.66	0.48
SO_4_^2–^	**0.73**	0.43		**0.71**	0.59
C_2_O_4_^2−^	**0.76**	0.45		0.69	**0.73**
Na^+^	0.13	0.08		− 0.03	0.02
NH_4_^+^	0.25	0.38		0.05	0.44
K^+^	0.58	0.09		**0.79**	0.25
Mg^2+^	0.39	0.24		0.25	0.21
Ca^2+^	0.50	− 0.06		0.52	0.18
OC	0.53	0.30		**0.80**	− 0.16
EC	0.42	0.02		0.67	0.03
Levoglucosan	0.30	− 0.37		0.63	− 0.51

Correlation coefficients higher than 0.7 are marked in bold.

OP^AA^ showed strong correlations with nitrate, sulfate, and oxalate during winter and with copper during the warm season. High correlation coefficients between AA measurements and Cu concentrations have been reported in many previous works (Fang et al. 2016; Janssen et al. 2014; Pietrogrande et al. 2018; Visentin et al. 2016). Since particulate Cu in urban environments is primarily associated with break wear emissions, this result points to traffic as a major source for OP^AA^ (Fang et al. 2016). A possible reason for the higher correlation coefficient observed in Elche during summer could be due to larger sulfate levels during this season (average SO_4_^2−^ concentrations were 1.21 and 2.67 µg m^−3^ during winter and summer, respectively). According to a study by Fang et al. (2017), high sulfate levels produce highly acidic conditions, favoring the dissolution of primary transition metals and contributing to OP levels. However, this hypothesis cannot be confirmed with our dataset because we have total Cu concentrations and not water-soluble Cu.

The elevated correlations between secondary species and OP^AA^ during winter differ from the outcomes of some previous works (Fang et al. 2016; Janssen et al. 2014) and cannot be explained on the basis of their toxicity, since there are no evidences of an association between components such as sulfate or nitrate and health effects (Ayres et al. 2008; Cassee et al. 2013). Therefore, these results may indicate a significant contribution of secondary processes to OP^AA^ during the cold season. The association between secondary aerosols and OP^AA^ measurements has been previously established by Fang et al. (2016). Indeed, they identified secondary processes as a major source for OP^AA^ in southeastern US, contributing 56% to the measured values. In contrast, other studies have found that biomass burning is a primary source for OP^AA^ (Borlaza et al. 2021). In the present study, OP^AA^ showed low *r* values with levoglucosan, considered as a reliable tracer of biomass burning (Vincenti et al. 2022), suggesting a limited contribution from this source to OP^AA^. This is not unexpected, given that biomass burning accounts for a small percentage of PM_10_ mass concentrations in the study area, as already mentioned.

The DTT assay was sensitive to more PM_10_ components than the AA method, as described in some studies (Fang et al. 2016), particularly during winter. Good correlations were found with non-exhaust (K, Fe, Cu, and Zn) and exhaust (EC and OC) traffic tracers, and with secondary components (NO_3_^−^, SO_4_^2−^, and C_2_O_4_^2−^). OP^DTT^ was also moderately correlated with levoglucosan and highly correlated with soluble potassium (K^+^) during the cold season, pointing to a significant influence of biomass burning emissions on the DTT activity. This is consistent with many previous findings showing that biomass burning is an important source for OP^DTT^ in winter (Bates et al. 2019; Borlaza et al. 2021; Calas et al. 2018; Fang et al. 2016; Giannossa et al. 2022).

The relationship between OP^DTT^ measurements and metallic elements (such as Fe, Cu, and Zn), derived mainly from vehicle wear (tires, brakes, discs…), has been established in a number of previously published works (Bates et al. 2019; Calas et al. 2018; Charrier and Anastasio 2012; Fang et al. 2016; Visentin et al. 2016). Transition metals induce DTT oxidation due to their ability to generate ROS, like H_2_O_2_ or even HO· radicals, by Fenton reactions (Bates et al. 2019; Charrier and Anastasio 2012; Jiang et al. 2019).

The organic fraction, associated with road traffic and biomass burning emissions, has also been identified as a primary contributor to the DTT activity (Cheng et al. 2021; Fang et al. 2016), especially catalytic redox-active compounds like quinones and water-soluble organic carbon (Bates et al. 2019; Charrier and Anastasio 2012; Jiang et al. 2019; Rao et al. 2020). The statistically significant correlations found in the present study between OP^DTT^ and levoglucosan (*p*-value < 0.01), as well as K^+^, point to a certain contribution from biomass burning to the DTT activity during the cold season, as already mentioned.

Secondary inorganic ions (namely sulfate and nitrate) are inactive in the DTT assay (Rao et al. 2020). Despite this, OP^DTT^ was strongly correlated with these species during the cold season. Previous research has also found an association between DTT measurements and secondary inorganic components (Cheng et al. 2021; Fang et al. 2016; Verma et al. 2009). These outcomes could be explained considering these components as indicators of secondary processes (Fang et al. 2016; Verma et al. 2009). Another possible reason for the correlation between the DTT activity and NO_3_^−^ levels could be that nitrate precursors are primarily emitted from combustion sources such as traffic and biomass burning (Giannossa et al. 2022).

During summer, correlations between OP^DTT^ and chemical components were weaker than in winter, with the exception of oxalate, which probably indicates that the relative contribution of secondary processes to the DTT activity was higher during the warm season.

The above results suggest that OP^DTT^ could eventually be a better metric of the biological reactivity of PM than OP^AA^, since it is sensitive to other PM_10_ components besides transition metals, such as OC and EC, that has been linked with adverse effects on human health (Molina et al. 2020). However, this is still an open issue that requires further research. Indeed, contrasting results have been reported in the literature. For instance, Cervellati et al. (2020) and Kelly et al. (2011) found that the AA assay is also sensitive to redox-active quinones, formed from the oxidation of aromatic compounds in the atmosphere. Instead, other works indicate that the DTT assay is the only method that has shown a positive association with specific health outcomes, while no relationship between the AA method and health effects has been stablished in human studies (Øvrevik 2019).

### Intrinsic oxidative potential

The intrinsic values of the AA and DTT activities reflect the oxidative potential of PM_10_ per unit mass ($\mathrm{OP}_{\mathrm m}^{\mathrm{AA}}\;\mathrm{and}\;\mathrm{OP}_{\mathrm m}^{\mathrm{DTT}}$, respectively) and are expressed in units of nmol min^−1^ µg^−1^. Table 4 shows intrinsic OP values measured by both assays during the study period.

**Table 4:** OP values normalized by PM_10_ mass (nmol min^−1^ µg^−1^) averaged for the whole period, summer and winter (± standard deviation)

	Mean	Winter	Summer
${\mathrm{OP}}_{\mathrm{m}}^{\mathrm{AA}}$	0.036 ± 0.019	0.029 ± 0.018	0.043 ± 0.017
${\mathrm{OP}}_{\mathrm{m}}^{\mathrm{DTT}}$	0.014 ± 0.007	0.018 ± 0.008	0.011 ± 0.004

*Differences between summer and winter averages were statistically significant (Student’s *t*-test, *p* < 0.05).

The values obtained by the two methods were larger than those found at a suburban site in southern Italy (${\mathrm{OP}}_{\mathrm{m}}^{\mathrm{AA}}$ = 0.009 nmol min^−1^ µg^−1^, ${\mathrm{OP}}_{\mathrm{m}}^{\mathrm{DTT}}$ = 0.008 nmol min^−1^ µg^−1^; Pietrogrande et al. 2018). The average ${\mathrm{OP}}_{\mathrm{m}}^{\mathrm{DTT}}$ was also slightly higher than the values reported for the same PM fraction in Riyadh (Saudi Arabia;${\mathrm{OP}}_{\mathrm{m}}^{\mathrm{DTT}}$ = 0.009 and 0.013 nmol min^−1^ µg^−1^ during dust and non-dust periods, respectively; Farahani et al. 2022). The intrinsic DTT activity was, however, significantly lower than the values measured for PM_2.5_ in more populated and polluted cities such as Milan (0.065 nmol min^−1^ µg^−1^), Athens (0.049 nmol min^−1^ µg^−1^) and Los Angeles (0.028 nmol min^−1^ µg^−1^) (Farahani et al. 2022), which indicates that atmospheric aerosols in these urban areas contain more toxic components.

A linear regression analysis between the intrinsic oxidative potential and the mass fraction of chemical components (amount of each component per unit mass of PM_10_) was performed. The results differed from those obtained for volume-normalized OP values. ${\mathrm{OP}}_{\mathrm{m}}^{\mathrm{AA}}$ only showed a strong correlation with Cu during the summer season (*r* = 0.92). On the other hand, ${\mathrm{OP}}_{\mathrm{m}}^{\mathrm{DTT}}$ was highly correlated with Cu (*r* = 0.70) and oxalate (*r* = 0.64) during summer, and with Cu (*r* = 0.75) and OC (*r* = 0.77) during the cold season. A moderate correlation coefficient was obtained between ${\mathrm{OP}}_{\mathrm{m}}^{\mathrm{DTT}}$ and levoglucosan in winter (*r* = 0.50).

The differences in the results of the correlation analysis between mass-normalized and volume-normalized OP values could be due to two reasons: (1) although some PM_10_ components may have a low capability to induce oxidative activity (i.e., they have a limited impact on the OP per unit mass), their higher atmospheric concentrations result in a greater contribution to volume-normalized OP values. For instance, ${\mathrm{OP}}_{\mathrm{m}}^{\mathrm{AA}}$ did not correlate with major secondary species (e.g., nitrate and sulfate), whereas volume-normalized OP^AA^ values showed a good correlation with these components during the summer season (see “Correlation of the oxidative potential with PM10 chemical components”). This could be attributed to the higher atmospheric concentrations of secondary species compared to other PM components (Table S1, Supplementary Material), which results in a greater overall exposure to redox-active secondary aerosols. Equivalent results were reported by Fang et al. (2016) for PM_2.5_ samples collected in Atlanta. For example, they found that traffic had a higher DTT intrinsic activity than biomass burning; however, biomass burning was the largest contributor to the volume-normalized DTT activity due to the strength of this source during the study period. (2) The larger oxidative potential of some species per unit mass may mask the capability of other PM_10_ components to induce redox reactions (Farahani et al. 2022). For example, the greater potency of Cu on a per unit mass basis in generating redox activity for the AA assay compared to secondary ions or carbonaceous species may have led to low *r* values between AA and the mass fraction of these components.

## Conclusions

The OP^AA^ and OP^DTT^ measured at an urban site in the western Mediterranean showed an opposite seasonal trend, although PM_10_ concentrations were similar during the cold and warm seasons. AA activity was greater in summer compared to winter, while DTT activity was higher in winter than in summer. This is most likely due to the different sensitivity of both assays to PM_10_ components and to seasonal changes in the chemical composition of PM_10_ samples. In fact, the AA reactivity (expressed in nmol min^−1^ m^−3^) was strongly correlated with Cu in summer and with secondary species (NO_3_^−^, SO_4_^2−^, and C_2_O_4_^2−^) during the cold season, while the DTT assay showed moderate-to-good correlation coefficients with a wide range of chemical components during winter, such as transition metals (Fe, Cu, Zn), secondary species (NO_3_^−^, SO_4_^2−^, and C_2_O_4_^2−^), and carbonaceous components (OC, EC, levoglucosan). These findings suggest that particles emitted from vehicular abrasion and secondary aerosols are the main contributors to the AA activity, whereas the OP^DTT^ is linked to combustion sources (traffic and biomass burning), non-exhaust vehicle emissions, and secondary processes. These results, which are in agreement with those of previous works, show that the DTT assay is sensitive to more PM_10_ components than the AA method, including combustion-related compounds known by their particularly serious health effects. Therefore, the OP^DTT^ could be considered as a better proxy for assessing the eventual toxicity of atmospheric aerosols than the OP^AA^. However, further research is needed to implement the use of the oxidative potential (either measured by one assay or as a combination of different assays) as an exposure metric for atmospheric PM in epidemiological studies.

## Supplementary Information

Below is the link to the electronic supplementary material.

Supplementary file1 (DOCX 15 KB)

## Data Availability

The datasets generated and analyzed during the current study are available from the corresponding author on reasonable request.
