# Supplementary material for: Sensitivity of PM10 oxidative potential to aerosol chemical composition at a Mediterranean urban site: ascorbic acid versus dithiothreitol measurements
Source: Air Qual Atmos Health. 2023 Mar 13;16(6):1165–72. doi: 10.1007/s11869-023-01332-1 (PMC10009354; doi:10.1007/s11869-023-01332-1)
Supplement: Supplementary file 1 — Supplementary file1 (DOCX 15 KB) [file 11869_2023_1332_MOESM1_ESM.docx]

**Table S1** Average concentrations and standard deviations (SD) of PM_10_ chemical components in Elche during summer and winter

|  | Winter |  |  | Summer |  |
| --- | --- | --- | --- | --- | --- |
|  | Average | SD |  | Average | SD |
| K (ng m^−3^) | 224 | 118 |  | 296 | 185 |
| Ti | 25 | 10 |  | 46 | 30 |
| Mn | 11 | 3 |  | 20 | 14 |
| Fe | 361 | 181 |  | 439 | 209 |
| Cu | 16 | 7 |  | 16 | 5 |
| Zn | 28 | 15 |  | 22 | 11 |
| Levoglucosan | 68 | 47 |  | 15 | 13 |
| Cl^–^ (µg m^−3^) | 0.47 | 0.52 |  | 0.95 | 1.10 |
| NO_3_^–^ | 1.85 | 1.90 |  | 1.95 | 1.08 |
| SO_4_^2–^ | 1.21 | 0.85 |  | 2.67 | 1.40 |
| C_2_O_4_^2–^ | 0.14 | 0.09 |  | 0.27 | 0.14 |
| Na^+^ | 0.45 | 0.41 |  | 1.31 | 0.75 |
| NH_4_^+^ | 0.12 | 0.25 |  | 0.13 | 0.16 |
| K^+^ | 0.26 | 0.16 |  | 0.15 | 0.09 |
| Mg^2+^ | 0.08 | 0.06 |  | 0.21 | 0.10 |
| Ca^2+^ | 1.89 | 0.77 |  | 1.74 | 0.80 |
| OC | 5.49 | 1.87 |  | 4.35 | 0.78 |
| EC | 1.21 | 0.56 |  | 1.09 | 0.23 |
